# Supplementary material for: Network topology and movement cost, not updating mechanism, determine the evolution of cooperation in mobile structured populations
Source: PLoS One. 2023 Aug 1;18(8):e0289366. doi: 10.1371/journal.pone.0289366 (PMC10393168; doi:10.1371/journal.pone.0289366)
Supplement: S1 File — Presentation and analysis of the data obtained with exploration phase length set at T = 1, under the two dynamics BDB and DBB, and different values of reward c. (PDF) [file pone.0289366.s001.pdf]

Supplementary Material:
Network topology and movement cost, not updating mechanism, determine the evolution of cooperation in mobile structured populations

Diogo L. Pires[1], Igor V. Erovenko[2], and Mark Broom[1]

[1]Department of Mathematics, City, University of London, Northampton Square, London EC1V 0HB, UK
[2]Department of Mathematics and Statistics, University of North Carolina at Greensboro, Greensboro, NC 27402, USA

July 5, 2023

We extend the analysis of the evolutionary outcomes obtained under the Markov movement model outlined in the main document of this paper. We focus on identifying if the similarity of qualitative outcomes between different evolutionary dynamics holds when we reduce the exploration phase length to the limiting value $T = 1$. We also assess the impact of considering different values of reward c.

In this setting, assortative behaviour is suppressed because individuals do not have iterated movement decisions. The isolated evolution of interactive strategies can be considered by fixing the movement strategies of residents and mutants, in which case the model becomes similar to an independent movement model such as the territorial raider one presented in [1, 2]. As will be shown, similar results to the ones obtained in that model and under static interaction networks [3] are recovered.

In the following tables 1, 2, and 3, we present the results obtained for the fixation probability of one cooperator on a population of defectors, under the same three topologies (complete, circle and star networks, respectively), for the two distinct dynamics BDB and DBB, and two different mobility scenarios.

Under co-evolved mobility, we consider the fixation probability of the fittest mutant cooperator on defectors with optimal staying propensities, a probability which is essential to the analysis under rare interaction mutations. Once again, the optimal staying propensity of resident defectors is $\alpha = 0.99$. The fittest mutant cooperators have the staying propensity that maximises their fixation probability on those defectors. This is the value represented in parenthesis, together with each fixation probability under co-evolved mobility.

Under fixed mobility, we consider that both resident defectors and mutant cooperators have the same staying propensity, which, with the purpose of simplifying our search, we have considered to be the one held by the fittest mutant cooperators under co-evolved mobility.

These results were obtained based on $100,000$ simulation trials for each combination of parameters. The estimation of the standard deviation is provided in [4]. We use the following parameter values: $N = 50$, $\lambda = 0.1$, $S = 0.03$, $c = 0.04$, $T = 1$, and we considered

the following different values of $v = 0.08, 0.4, 2, 8$. Note that fixation probabilities are compared to their value under neutral selection, which corresponds to $1/N = 0.02$. Probabilities higher than this value are highlighted in the tables.

For an extensive analysis of the parameter space, we refer to [4], on whose supplementary material the impact of different values of reward-to-cost ratio and exploration time is assessed considering only the BDB dynamics.

	Co-evolved mobility		Fixed mobility	
	BDB	DBB	BDB	DBB
$v = 0.08$	0.0063 (0.99)	0.0015 (0.99)	0.0063	0.0015
$v = 0.4$	0.0065 (0.9)	0.0021 (0.8)	0.0057	0.0022
$v = 2$	0.0170 (0.8)	0.0159 (0.6)	0.0050	0.0019
$v = 8$	**0.0202** (0.9)	**0.0323** (0.4)	0.0018	0.0003

Table 1: Fixation probabilities of cooperators under a complete network, for two distinct mobility scenarios, and different reward values and evolutionary dynamics. The value included in parenthesis together with each fixation probability value, under co-evolved mobility, corresponds to the calculated staying propensity of the fittest mutant cooperators. Resident defectors under co-evolved mobility were considered to use their optimal staying propensity of 0.99. Fixation probabilities under fixed mobility were calculated using the same staying propensity for both mutants and defectors as the obtained for the corresponding fittest mutants under co-evolved mobility. Note that fixation probabilities are compared to their value under neutral selection, which is 0.02 – probabilities higher than this value are highlighted.

In table 1, we observe that for reward values up to $v = 2$ in the complete network, mutant cooperators do not fixate above neutrality both under the co-evolution of movement strategies and when these are fixed at the same value for resident defectors. In comparison, the main results of this paper show that under longer exploration phases of $T = 10$ and under $v = 0.4$, cooperators fixate under all dynamics for this movement cost $\lambda = 0.1$.

When the reward value is high enough ($v = 8$), co-evolving mobility allows for the successful fixation of cooperators. However, under fixed mobility, we observe that the fixation of cooperators decreases for the highest values of the reward. The fact that it never reaches the neutral fixation threshold is in accordance with the proposition that under $T = 1$ and fixed mobility, assortative behaviour vanishes, and only the spatial viscosity of the evolutionary process described in [3], and also observed in [2], can sustain cooperation. Viscosity is not present in complete networks, as all individuals are connected, hence the lack of success of cooperators under fixed mobility.

The differences between dynamics are smaller under this topology, and are mainly related to the overall effect of amplification of selection described in the main results from this paper.

Table 2 shows the results obtained under circle networks. These are fairly similar to the ones from complete networks under co-evolved mobility, but they hold key differences under fixed mobility. In the later, we observe that fixation probabilities under the BDB dynamics are consistently below neutrality, while under the DBB they increase considerably for larger reward values, reaching values above that threshold for both $v = 2$ and $v = 8$. This shows that under $T = 1$, and in the absence of co-evolving mobility, the viscosity of the process can still allow for the fixation of cooperation. This is again in agreement with the conclusions from [3], that if the average degree of a network is lower than (a function)

	Co-evolved mobility		Fixed mobility	
	BDB	DBB	BDB	DBB
$v = 0.08$	0.0062 (0.99)	0.0015 (0.99)	0.0062	0.0015
$v = 0.4$	0.0070 (0.9)	0.0034 (0.8)	0.0063	0.0040
$v = 2$	**0.0285** (0.8)	**0.0564** (0.7)	0.0062	**0.0221**
$v = 8$	**0.0622** (0.8)	**0.1040** (0.7)	0.0067	**0.0378**

Table 2: Fixation probabilities of cooperators under a circle network. Other information as in the caption of table 1.

of the reward-to-cost ratio, cooperation can evolve under some dynamics. Here we further show that the distinct nature of the BDB and DBB dynamics is recovered when there is no co-evolved assortative behaviour. This difference should be associated with the network viscosity of the process but not with the later mechanism.

	Co-evolved mobility		Fixed mobility	
	BDB	DBB	BDB	DBB
$v = 0.08$	0.0064 (0.99)	0.0022 (0.99)	0.0064	0.0022
$v = 0.4$	0.0105 (0.7)	**0.0288** (0.4)	0.0059	0.0032
$v = 2$	**0.0530** (0.5)	**0.1731** (0.01)	0.0049	0.0043
$v = 8$	**0.0760** (0.6)	**0.2551** (0.01)	0.0023	0.0027

Table 3: Fixation probabilities of cooperators under a star network. More information included in the caption of table 1.

Finally, the results obtained under star networks hold similarities with to the ones obtained under complete networks. Under co-evolved mobility, cooperation fixates under both evolutionary dynamics for high enough rewards. The minimum value for which it happens is lower under the DBB dynamics, and, once again, these dynamics amplify selection and allow for cooperators to fixate with high probabilities. Under fixed mobility, this network leads to fixation probabilities as low as under complete networks. This is a highly centralised network, where all individuals can potentially meet (in the centre), therefore corresponding to a highly connected interactive structure, under which viscosity is no longer present [3], and cooperation cannot evolve under $T = 1$ without co-evolved mobility.

In summary, we observed that strictly limiting exploration phases to $T = 1$, co-evolving staying propensities and network viscosity can still allow cooperation to fixate. The later is related to the mechanism analysed in the context of evolutionary games for the first time in [3]. Here we recover a result similar to the original rule stated there: cooperation can evolve only in networks with low enough degree, far from complete, and under particular evolutionary dynamics. These results are relevant for the analysis we perform in the paper, as they show that the fundamental differences between some of these dynamics come from their relation to the viscosity of evolutionary processes on networks, and are often not reflected in the presence of co-evolving assortative behaviour.

References

[1] Broom M, Lafaye C, Pattni K, Rychtář J. A study of the dynamics of multi-player games on small networks using territorial interactions. Journal of Mathematical Biology. 2015

3;71(6-7):1551–1574.

[2] Pattni K, Broom M, Rychtář J. Evolutionary dynamics and the evolution of multiplayer cooperation in a subdivided population. Journal of Theoretical Biology. 2017 9;429:105–115.

[3] Ohtsuki H, Hauert C, Lieberman E, Nowak MA. A simple rule for the evolution of cooperation on graphs and social networks. Nature. 2006 5;441(7092):502–505.

[4] Erovenko IV, Bauer J, Broom M, Pattni K, Rychtář J. The effect of network topology on optimal exploration strategies and the evolution of cooperation in a mobile population. Proceedings of the Royal Society A: Mathematical, Physical and Engineering Sciences. 2019;475(2230).
