# Supplementary material for: Network topology and movement cost, not updating mechanism, determine the evolution of cooperation in mobile structured populations
Source: PLoS One. 2023 Aug 1;18(8):e0289366. doi: 10.1371/journal.pone.0289366 (PMC10393168; doi:10.1371/journal.pone.0289366)
Supplement: Attachment — Submitted filename: response.pdf [file pone.0289366.s002.pdf]

Response to Reviewers

July 5, 2023

We would like to thank the reviewers for reading our manuscript very carefully and making numerous excellent suggestions that allowed us to improve it significantly. Below we answer each comment to the best of our ability. Alterations and additions to the original manuscript were included in blue text in the newly submitted document. A supplementary material document was now included with new simulated data and its analysis.

Reviewer 1

1. "The model is nice because the framework is generally described that is suitable for a theoretical analysis. But, I see their model extremely specific than a simple spatial version of PGG with a mobile option. For instance, what they introduced for the exploration phase and evolutionary one seems quite specific, neither common with the previous models as above nor generally observed in a real society at all. I do believe that the authors need more deliberate explanation of why their model can be justified."

   Response: We have now added a detailed explanation justifying our specific model and its connection to the original evolutionary graph theory models, in four new paragraphs, just before the last paragraph of the Introduction. In particular we discuss evolutionary graph theory, its connection to the simplest case within our framework, the independent model, and why we believe that our extension to a mobile population (including with the distinct phases) is logical and appropriate.

2. "Following to the previous item; #1, I thing that the statement; their finding is very much contrasting to the precursors' results that cooperation can only emerge under some update rules and if the average degree is low, should be more deliberately proved. I totally agree that this is the most important finding in the present study, which (literally) attracts many audience in the arena. But I'm skeptical whether this statement was fairly proved or not. It's because the most of the previous studies in view of a spatial version PGG with a mobile option are different from theirs that can be said too specific as abovementioned."

   Response: The statement from the reviewer "the precursors' results that cooperation can only emerge under some update rules and if the average degree is low" describes the main results observed in [1] for static interaction networks. See the following sentence quoted from that paper:

   "At first, we will study the following update rule for evolutionary dynamics (Fig 1): in each time step, a random individual is chosen to die, and the neighbors compete

for the empty site proportional to their fitness. We call this mechanism ‘death-birth’ updating, because first there is a death event followed by a birth. (...)

In particular, we find that cooperators have a fixation probability greater than $1/N$ and defectors have a fixation probability less than $1/N$, if $b/c > k$. The ratio of benefit to cost of the altruistic act has to exceed the degree, k, which is given by the number of neighbors per individual. This condition is derived for weak selection and under the assumption that the population size, N, is much larger than the degree, k. (...)

There are also update rules, where selection can never favor cooperators. For example, let us consider ‘birth-death’ updating: at each time step an individual is selected for reproduction proportional to fitness, and the offspring replaces a randomly chosen neighbor. In this case, selection always favors defectors...”

To clarify what we meant with the degree has to be low ($k < b/c$ and $k << N$), we have rephrased that statement in the abstract and in the introduction (section 1), and made it closer to what is in the discussion (section 4). Now it says that it has to be “lower than the reward-to-cost ratio and the network far from complete”.

Additionally, it is also observed in [2] that cooperation has much more difficulty evolving under BDB in the territorial raider model as it is said in the following quote from that paper:

“For DBD dynamics, the defectors do better than cooperators regardless of the population structure. However, for DBB dynamics, cooperators are favoured over defectors for certain population structures. In particular, these structures that favour cooperators contain small subpopulations, ideally of two individuals.”

These observations are extensively described and discussed in section 4, and we added a few more lines to highlight these central aspects of our analysis

From our general results we show that cooperation evolves the most easily under complete networks and for all evolutionary dynamics comparing all region figures, it is clear that the ones related to the complete network—figures 1 and 7—are the ones with the largest regions where cooperation is stable. The reviewer suggests that this difference with the previous models may come from the differences introduced by our model. In the paper, we argue that they come specifically from the conditional movement in our model and from the assortative behaviour that co-evolves in the population because of it. This is further confirmed by the fact that the territorial raider model in [2], similar to the one we use in this paper but with independent movement, seems to hold similar results to the ones from static networks [1], as quoted above. We develop the discussion about this in section 4 and we add new references to papers where assortative behaviour, co-evolving or not, is shown to sustain cooperation.

To further address this point, we added results from new simulations onto a new supplementary material document with reduced exploration phases $T = 1$. In this limit, individuals don’t have time to form assortative behaviour. We show that by taking that away, the differences between dynamics are substantially increased, and by taking away the co-evolution of strategies, we seem to recover that cooperation only evolves under the DBB (and not under the BDB) dynamics, for highly structured networks with low degree of nodes (circle), and for large enough reward-to-cost-ratios. We incorporate an analysis of those results and extended the previous comparative analysis with the literature in section 4 (Discussion).

3. "The authors mainly varied the mobile cost, the population besides the evolutionary dynamics and network topology. That's fine. But everyone unequivocally perceives that there is another important model parameter of which sensitivity should be carefully explored. That is the dilemma strength of PGG. In the present model, the dilemma strength is influenced by v, c and the baseline income; +1. By referring to the universal dilemma strength; c/v relates to the dilemma strength, while v/c (for which, in the conventional PGG, b/c is adopted) indicates the dilemma weakness. I guess that they did fix the dilemma strength (dilemma weakness). They need further discussion on this point with sharing another series of results. When they add this, they should reference to the universal concept of dilemma strength for both 2-player & 2-strategy games (including PD, Snowdrift, Stag Hunt etc) and multi-player & 2-staretgy including PGG with citation of the relevant literature; for instance, (i) Social efficiency deficit deciphers social dilemmas, Scientific Reports 10, 16092, 2020, (ii) Sociophysics Approach to Epidemics, Springer, 2021."

   Response: An extension of the analysis of results from a Markov model under the BDB dynamics to other region of the payoff parameter space is presented in the supplementary material of [3]. That was mainly obtained by varying the value of the reward-to-cost ratio v/c, which can be interpreted as the inverse of a dilemma strength value alternative to the universally scaled one from pairwise games defined in [4]. A higher value of v/c represents a lower dilemma strength and a higher social efficiency deficit [5], i.e., the potential gains of moving the population from its Nash equilibrium (everyone defects) to the collective optimal state (everyone cooperates). This quantifies how easy it is for the dilemma to be solved (i.e. relaxed) through additional overlapping mechanism [6]. The analysis presented in [3] showed that higher ratios generally led to higher mobility and more co-evolved cooperation across the parameter space under the three topologies, i.e. the dilemma becomes easier to relax [4].

   Even if the impact of changing v/c has been studied already in Markov models, we recognise that the manuscript as it was lacked an analysis of the impact of choosing a particular value of the reward-to-cost ratio. To tackle this, we did the following:

   – We added new results from simulations in the supplementary material, where we vary the reward-to-cost ratio v/c from 2 to 200 ($v = 0.08$ to $v = 8$ for fixed $c = 0.04$) for two different dynamics (BDB and DBB) and a different length of the exploration phase ($T = 1$), under which the current Markov model is reduced to a simpler limit. In this particular case the differences between the dynamics reemerge, particularly if we consider constant staying propensities.

   – We have added a few points of analysis to section 4 (now named "Discussion" following one of the points from reviewer # 2) where we comment on the results from [3] where v/c was varied, which was lacking so far. We also comment on the new simulations run for different values of v/c under $T = 1$ and two different dynamics.

   – We have added a point of discussion in Model section when we introduce the game, expanding on the fact that having an excludable public good makes our game a social dilemma regardless of the values of v and c, whereas a non-excludable version of the game is only a social dilemma for all group sizes if $v/c < 2$ (see definition in [7]). We have also commented on the fact that the reward-to-cost ratio (v/c) under this dilemma is similar to the inverse of the universally scaled dilemma strength [4], and

relate this to the alternative measure of social efficiency deficit [5].

Reviewer 2

1. "In the manuscript, the definition of fitness in equation 7 and the game dynamics listed in Table 1 are shown to be independent of the selection strength, indicating that the results were obtained under strong selection scenarios. However, it is worth considering the potential impact of weak selection on the evolutionary outcomes. Many existing studies have highlighted the significance of weak selection in shaping evolutionary dynamics. Therefore, I would like to inquire about the robustness of the presented results with respect to variations in the selection strength."

   Response: Studying the outcome of evolutionary processes under the limit of weak selection is indeed a common practice in the context of evolutionary game-theoretic approaches. This is mainly due to the mathematical tractability of that limit, and the fact that it allows an analysis in cases where strong selection does not. This limit is valuable because it provides simple mathematical rules or insights which are then extrapolated (or sometimes demonstrated, e.g., through simulation) to be valid under regimes of stronger selection.

   However, due to the relative complexity of the model we used in the present manuscript, studying this limit analytically is not possible. This is the reason why we have not considered it and also why we have not parameterised the intensity of selection explicitly—even though our addition of a background payoff of 1 could indirectly be used to explore such limits.

   Considering this limit and resorting to simulations would be counterproductive, as not only no mathematical advantage would be gained from it, but the fixation probabilities would be close to neutral ($1/N$), and any small advantages of certain strategies would be small and subject to the dominating noise of stochastic fluctuations. This is the main reason why there are not examples in the literature on evolutionary games where the weak selection limit is simulated.

   Nonetheless, an alternative extension of the analysis of Markov model results to other regions of the payoff parameter space is presented in the supplementary material of [3]. This was mainly obtained by varying the value of the reward-to-cost ratio v/c, also known as the dilemma weakness. That analysis showed that higher ratios generally led to higher mobility and more co-evolved cooperation, under the three topologies. Additionally, we have added new results from simulations to a Supplementary Material document, where we study the effect of reducing the exploration phase, while at the same time assessing the impact of the reward-to-cost ratio on the observed results.

   We now discuss some of these aspects in section 2.2 (Model – Multiplayer Game), section 2.3 (Model – Evolutionary Dynamics), and briefly on section 4 (Discussion), as mentioned above.

2. "The authors chose three types of networks, namely the complete graph, circle network, and star network. However, it would be helpful to clarify whether these networks are representative in the current study. In the literature, it is widely recognized that cooperators can thrive in social networks due to network reciprocity, where cooperators tend to form cohesive clusters to support one another. Therefore, I suggest

considering the inclusion of a regular lattice as it is often considered more representative. Additionally, by adjusting the short-cutting probability of links in a square lattice with a degree of 8, it is possible to generate various homogeneous networks with different clustering coefficients."

Response: Our primary motivation for considering the complete, circle, and star networks is that the current Markov model was investigated in detail for these networks in [3] for the BDB updating mechanism. The current paper is an extension of that study which focuses on comparing the results obtained under diverse updating mechanisms. These networks hold extreme values of network metrics such as the cluster coefficient and the degree variance (i.e, centralisation of the network), which were hypothesised to determine some of the dynamics occurring on these networks. We did not include other networks because the associated simulations are computationally expensive, and the complexity of the model allows few analytical shortcuts. For example, it took us approximately half a year of intensive computations on HPC clusters to obtain the results presented in this paper. We did conduct an investigation of this evolutionary process on various random networks in a separate paper [8], which is currently under revision. Those random networks represent a wider variety of topologies compared to the extreme cases of complete, circle, and star networks. Our main finding was that the network topology affects the outcomes of the evolutionary process only for networks of small average degree. Once the degree becomes sufficiently high, the outcomes match those for the complete graph. However, the actual value of the degree when this happens is much smaller than that of the complete graph.

We have added a paragraph in the Discussion section to address this issue.

3. "Section of Discussion. The discussion section appears to be relatively brief and lacks a thorough comparison with existing studies that argue cooperation can only emerge under specific update rules and when the average degree is low. To enhance the discussion, I suggest providing a more comprehensive analysis and explaining the divergence between the findings presented in this study and the existing literature. This will help readers understand the novelty and significance of the results obtained."

   Response: Section 3.3, previously named "Discussion", focuses on the topological aspects directly observed from the comparison of the results obtained in the two mutation scenarios. We perform a thorough comparison of our results to existing studies on section 4, which was previously named "Conclusions". This is described in diverse parts of the text, e.g., the beginning of section 3, and the end of section 3.3. As suggested by the reviewers' enquiry, the previous organisation of these two sections 3.3 and 4 might leave the reader wondering about the completeness of the discussion analysis. To address such a possibility, we have performed the following actions:

   – We have renamed the sections 3.3 and 4 to "The two mutation scenarios in comparison" and "Discussion" respectively. The new organisation highlights that in section 3.3 we analyse only aspects related to features observed in the two mutation scenarios, while it is in section 4 that the core of the analysis and literature comparison is presented. We have rephrased some of the descriptions of the content (see beginning of section 3), in order to highlight the fact that the core of the analysis of results and literature comparison is done in section 4.

   – We have extended the literature comparison present in section 4, clarifying and highlighting some of the differences with previous evolutionary graph theory models

(also added a few paragraphs related to this in section 1 based on the comment 1 left by reviewer 1) and adding new references of assortative behaviour models.

– We have restructured section 4 to highlight the parts where where we perform a "comprehensive analysis" and the paragraphs where we explain, between other things, "the divergence between the findings presented in this study and the existing literature". We additionally add new simulation results to a supplementary material document, whose analysis further shows that the differences with previous models tend to vanish when we consider the simplifying limit where there is no co-evolving assortative behaviour.

References

[1] Ohtsuki H, Hauert C, Lieberman E, Nowak MA. A simple rule for the evolution of cooperation on graphs and social networks. Nature. 2006 5;441(7092):502–505.

[2] Pattni K, Broom M, Rychtář J. Evolutionary dynamics and the evolution of multiplayer cooperation in a subdivided population. Journal of Theoretical Biology. 2017 9;429:105–115.

[3] Erovenko IV, Bauer J, Broom M, Pattni K, Rychtář J. The effect of network topology on optimal exploration strategies and the evolution of cooperation in a mobile population. Proceedings of the Royal Society A: Mathematical, Physical and Engineering Sciences. 2019;475(2230).

[4] Ito H, Tanimoto J. Scaling the phase-planes of social dilemma strengths shows game-class changes in the five rules governing the evolution of cooperation. Royal Society Open Science. 2018;5(10):181085. Available from: https://royalsocietypublishing.org/doi/abs/10.1098/rsos.181085.

[5] Arefin MR, Kabir KMA, Jusup M, Ito H, Tanimoto J. Social efficiency deficit deciphers social dilemmas. Scientific Reports. 2020 12;10(1):16092.

[6] Tanimoto J. Sociophysics Approach to Epidemics. Singapore: Springer; 2021. Available from: http://www.springer.com/series/11930.

[7] Broom M, Pattni K, Rychtář J. Generalized Social Dilemmas: The Evolution of Cooperation in Populations with Variable Group Size. Bulletin of Mathematical Biology. 2019 11;81(11):4643–4674.

[8] Erovenko IV, Broom M. The evolution of cooperation in a mobile population on random networks: Network topology matters only for low-degree networks. Submitted. 2023.
